# On the resilience of health systems: A methodological exploration across countries in the WHO African Region

**DOI:** 10.1371/journal.pone.0261904

**Published:** 2022-02-07

**Authors:** Humphrey Cyprian Karamagi, Regina Titi-Ofei, Hillary Kipchumba Kipruto, Aminata Benitou-Wahebine Seydi, Benson Droti, Ambrose Talisuna, Benjamin Tsofa, Sohel Saikat, Gerard Schmets, Edwine Barasa, Prosper Tumusiime, Lindiwe Makubalo, Joseph Waogodo Cabore, Matshidiso Moeti

**Affiliations:** 1 Data, Analytics and Knowledge Management - WHO Regional Office for Africa, Brazzaville, Congo; 2 Health Information Systems team - WHO Regional Office for Africa, Brazzaville, Congo; 3 Emergency Preparedness and Response Cluster - WHO Regional Office for Africa, Brazzaville, Congo; 4 Health Policy and Systems Research Team - KEMRI Wellcome Trust Research Programme, Nairobi, Kenya; 5 Health Services Resilience Team - World Health Organization Headquarters, Geneva, Switzerland; 6 Primary Health Care Special Programme - World Health Organization Headquarters, Geneva, Switzerland; 7 Health Economics Research Unit, KEMRI - Wellcome Trust Research Programme, Nairobi, Kenya; 8 Health Systems Expert, Kampala, Uganda; 9 Assistant Regional Director, WHO Regional Office for Africa, Brazzaville, Congo; 10 Director of Program Management, WHO Regional Office for Africa, Brazzaville, Congo; 11 Regional Director, WHO Regional Office for Africa, Brazzaville, Congo; Njala University, SIERRA LEONE

## Abstract

The need for resilient health systems is recognized as important for the attainment of health outcomes, given the current shocks to health services. Resilience has been defined as the capacity to “prepare and effectively respond to crises; maintain core functions; and, informed by lessons learnt, reorganize if conditions require it”. There is however a recognized dichotomy between its conceptualization in literature, and its application in practice. We propose two mutually reinforcing categories of resilience, representing resilience targeted at potentially known shocks, and the inherent health system resilience, needed to respond to unpredictable shock events. We determined capacities for each of these categories, and explored this methodological proposition by computing country-specific scores against each capacity, for the 47 Member States of the WHO African Region. We assessed face validity of the computed index, to ensure derived values were representative of the different elements of resilience, and were predictive of health outcomes, and computed bias-corrected non-parametric confidence intervals of the emergency preparedness and response (EPR) and inherent system resilience (ISR) sub-indices, as well as the overall resilience index, using 1000 bootstrap replicates. We also explored the internal consistency and scale reliability of the index, by calculating Cronbach alphas for the various proposed capacities and their corresponding attributes. We computed overall resilience to be 48.4 out of a possible 100 in the 47 assessed countries, with generally lower levels of ISR. For ISR, the capacities were weakest for transformation capacity, followed by mobilization of resources, awareness of own capacities, self-regulation and finally diversity of services respectively. This paper aims to contribute to the growing body of empirical evidence on health systems and service resilience, which is of great importance to the functionality and performance of health systems, particularly in the context of COVID-19. It provides a methodological reflection for monitoring health system resilience, revealing areas of improvement in the provision of essential health services during shock events, and builds a case for the need for mechanisms, at country level, that address both specific and non-specific shocks to the health system, ultimately for the attainment of improved health outcomes.

## Introduction

Health systems are perceived to be functional when they are able to make available the services people need for their health and well-being. Disruptions to system functioning impede availability of services, as well as utilization [1]. The effects of these on overall health targets have been well documented, following outbreaks of new or re-emerging diseases, such as the 2014–16 Ebola Virus Disease (EVD) outbreak in West Africa [2, 3]. Health systems have shown limited capacity to absorb unexpected increases in service demand, driven by shock events- an issue documented not only with disease events in low income countries but even following natural disasters such as earthquakes in Japan [4]. This effect of shocks on systems and services, including the large impact on indirect deaths have been documented extensively following the 2014–16 West African Ebola Virus Disease (EVD) outbreak, and currently in the context of the COVID-19 pandemic [5, 6].

The occurrence of these system shocks have strengthened the case for building health systems resilience—defined as the ability to sustain provision of essential services even when subject to a disruption, [7–9]. However, the practical application of interventions to make systems resilient has so far not been clear [10]. In many countries, these interventions have focused on minimizing the disruptive event, through actions for “preparing for and effectively responding to crises; maintaining core functions when a crisis hits; and, informed by lessons learnt during the crisis, reorganise if conditions require it” [7, 9, 11–13].

Despite these heavy investments to integrate emergency preparedness and response actions into health systems across the African Region, the region’s health systems are still perceived to not be resilient enough to a broad range of shocks. The literature, particularly drawing lessons from recent epidemics, has emphasized the need to build health system qualities that better allow them to anticipate, absorb, adapt and transform in a manner that sustains delivery of essential services [8, 9]. These challenges of resilience are further aggravated by the wide dichotomy between published evidence and practice of resilience strengthening, as well as the limited empirical analysis needed to guide comprehensive planning for building effective health system resilience on the ground [10, 14, 15]. In this paper, we explore an approach to integrating the concepts and interpretations of resilience, recognizing the nascent nature of the conceptualization of health systems and services resilience, and thus contributing to this growing body of important evidence. We base this on information from countries in the WHO African Region, to facilitate better planning and monitoring of the health agenda in the region [16].

We explore resilience across four areas: (1) what range of events constitute shocks; (2) how these shocks affect the capacity to deliver services; (3) options for minimizing the effects of these shocks; and (4) capacities a system needs to have to minimize these shocks. The paper also proposes an approach to monitor and prioritize actions to improve the integrated capacities for resilience, using a cross-sectional data collection approach.

### Range of events that disrupt provision of essential services

Most literature relating to resilience is based on acute events such as EVD or COVID-19, and environmental and/or climatic events like floods or drought [14, 17]. There however exists a wider range of shocks, including more chronic shocks that call for ‘everyday resilience,’ given their sustained and nuanced nature [18–21]. We structure the full range of possible shock events in Table 1, based on the nature of their onset and classification.

**Table 1:** Shocks to health systems that hinder provision of essential services in WHO African Region.

	DISEASE EVENTS	ENVIRONMENTAL EVENTS	ECONOMIC EVENTS	POLITICAL EVENTS
**Acute: Sudden onset, and/or duration**	New/re-emerging disease event of a sudden onset, and/or expected shorter term duration, e.g., EVD, COVID-19	Sudden onset of changes in climate affecting health, e.g., floods, mudslides	Sudden fiscal event that changes available funding for health, e.g., unexpected donor withdrawal, oil price shocks	Political events forcing a sudden change in health direction, e.g., a coup, political insurgence
**Chronic: Gradual onset, and / or duration**	New/re-emerging disease event of a longer-term onset, and/or expected longer term duration, e.g., cholera outbreak, NCD burden	Effects of climate change events affecting health, e.g., drought	Progressive fiscal events changing available funding for health, e.g., progressively reduced donor confidence, less government health prioritization	Political events leading to slow, sustained change in health direction, e.g., due to imposed health stewards or to inadequate leadership capacity

Source: Author’s construction.

### How shock events affect capacity to deliver services

Shock events can either affect health directly—for instance floods killing people—or indirectly—for instance through disrupting provision of services. The nature of the direct effect is a function of the shock and its severity, while the indirect effect is a function of the existing context and capacity of the health system. The COVID-19 pandemic for instance, directly led to deaths due to COVID-19, and indirectly disrupted countries’ abilities to provide essential health, social and economic services, thus leading to excess deaths [22–24]. We postulate four different ways a health system may be affected by a given shock event: (1) it is unable to provide essential services it was providing before, as was seen in some areas and particularly inpatient services during the EVD outbreak in West Africa [25, 26]; (2) it is providing reduced essential services compared to what it was previously able to, usually targeting specific service functions [3, 27]; (3) it is able to sustain provision of essential services it was previously providing even as it responds to the shock event [28, 29]; or (4) there is no effect and the system reverts to its pre-shock status. Of these, only option 3 represents an optimal scenario of how a resilient system responds to a shock event—its ability to handle both services due to the shock event and simultaneously keep essential services uninterrupted, whilst positively transforming in the process [30].

This perspective goes beyond looking at resilience as a coping and reactive mechanism to a shock event [31–33], but equally emphasizes the need for strengthening systems to allow them to address a broad range of unpredictable shocks [11, 14, 18]. It is not only about embedding capacities to anticipate and manage potential emergencies and/or disasters [34], but in addition, should focus on capacities existing in the system that can be leveraged as and when the system faces any shock.

Resilience is thus not just the response and recovery from a specific shock. It needs to be interpreted as a process that facilitates transformation of the health system in a way that sustains its functioning even when affected by shocks, irrespective of their nature [30, 35, 36]. As an illustration, a house has, as core properties, a roof, doors and windows which can also provide some support against a broad range of non-specific shocks, such as extreme weather and/or thieves. However, if there is an increased risk of a given shock, the homeowner may reinforce the house for instance, with a lightening arrester for lightening protection, burglar proofing, a perimeter wall or guard dogs for thieves. The resilience of the home is contributed to by the structure for non-specific shocks together with the specific security measures targeting known potential shocks.

**Fig 1:**
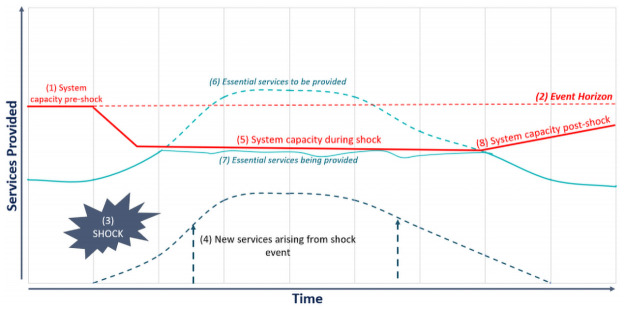
Effects of a shock event on provision of essential services.

We illustrate these two concepts in Fig 1 below. Health systems have a certain threshold of service provision capacity- we term this its ‘event horizon’. This capacity could be reduced by a shock event in two ways: (1) the shock event introduces new services not planned for, such as the care needed to treat Ebola during an outbreak, and (2) the existing capacity to provide services is diminished as they are severely disrupted by the shock event, and/or are diverted/repurposed to respond to the shock—for instance health workers getting sick, and/or diverted from providing antenatal care to emergency response services. The system is therefore not able to adequately provide services it was previously able to during the period of the shock.

The resilience capacities aim to either minimize the additional demands due to the shock event and/or enhance the system’s ability to function above its existing capacity. The shock event could lead to disease and death directly—such as persons ill or dying from COVID-19 –or indirectly—such as persons ill or dying because they cannot access other essential services [37]. The EPR capacities are needed to respond to predictable shock events as they are usually specific in nature (for instance, a cholera or meningitis outbreak), while the ISR capacities facilitate response to unpredictable events and enable system functioning above existing capacity (for instance the SARS-CoV-2 outbreak). A resilient system needs to exhibit both EPR and ISR.

### How we identify levels of EPR and ISR

The literature documents three approaches to collating information on emergency preparedness and response capacities across countries: the global health security (GHS) index [38], the IHR State Party Annual Reporting (SPAR) mechanism [39], and the Joint External Evaluation (JEE) process [40]. The GHS index is an outcome based global assessment, while the JEE and the SPAR are output based country assessments. The IHR SPAR scores are derived from annual country self-reporting on the capacity of attributes constructed from 13 components, while the JEE is an external expert assessment carried out every 4–5 years as part of the IHR Monitoring and Evaluation Framework. Given the periodicity, the focus on output capacity and the level of country ownership, the IHR SPAR process provides a reliable, country owned and transparent source of information for assessing emergency preparedness and response capacities. Furthermore, the IHR SPAR is the only international health emergency mechanism formally endorsed by all the 196 state parties, indicative of global and country-level buy-in and consensus on its utility in monitoring and reporting on capacity to respond to potential shock events. Studies have also shown a strong correlation between the IHR SPAR and JEE (23 of 24 SPAR indicators map either closely or directly with JEE indicators), which provides a peer-reviewed evaluation perspective through the IHR Monitoring and Evaluation Framework (IHRMEF). Thus, despite the criticisms related to the self-reporting nature of the IHR SPAR [41], its correlation with the IHR JEE which increases accountability, objectivity and transparency of the IHR processes contributes to the case made for its selection as the basis of EPR in this paper [42]. Furthermore, the global health security index still remains to have widespread buy-in, with ongoing debates on its methodological soundness [43].

The inherent system resilience capacities on the other hand have no similar consensus and are largely still the preserve of academic deliberations [10]. While this is useful from a conceptual perspective, a health decision maker needs to make normative choices and actions as they implement actions to improve ISR [44]. A large volume of this literature is descriptive—with an absence of normative and empirical guidance on how to translate this understanding into activities that can be planned for, measured and monitored in their totality [45]. This challenge is further noted by Biddle *et al*., who recognize the gap in quantitative measures on resilience, as well as a lack of clearly defined characteristics for measurement, pre-conditions and limits of the concept [14]. Furthermore, Razavi et al. also provide further justification of the need for assessing health system resilience. They state that “neither JEE nor SPAR may fully assess preparedness as other factors outside of IHR compliance such as political engagement and health system resilience will impact preparedness and response”.

As a starting point, Kruk et al. (2015) provide a description of what resilience may entail—these attributes being distinctly different from the EPR capacities [9]. We adopt, as a working approach, these descriptions as capacities of ISR. These descriptions are adopted, in lieu of other frameworks for assessing health service resilience, given its comprehensive effort to consolidate various research on health service resilience. Furthermore, Kruk et al.’s framework aligns with the WHO AFRO Framework for Health Systems Development [46], which has been endorsed by all 47 Ministers of Health of the WHO African Region at the 67^th^ Regional Committee of the WHO African Region [46]. This framework positions resilience as a key component of the functionality of health systems, together with other key dimensions of access to services, demand for services and quality of care [47, 48]. We thus use this framework as a basis of our empirical analysis, recognizing that there remains scope for improvement in sharpening the focus of various frameworks related to health system resilience.

The attributes in Kruk et al.’s framework include: (1) system awareness—how well the system knows its abilities and vulnerabilities; (2) diversity—the range of services the system can provide; (3) self-regulation—the ability of the system to make decisions on response to threats; (4) mobilization—the system’s ability to bring together resources to build a response; and (5) transformation—the system’s ability to learn from and apply lessons to future shocks.

**Fig 2:**
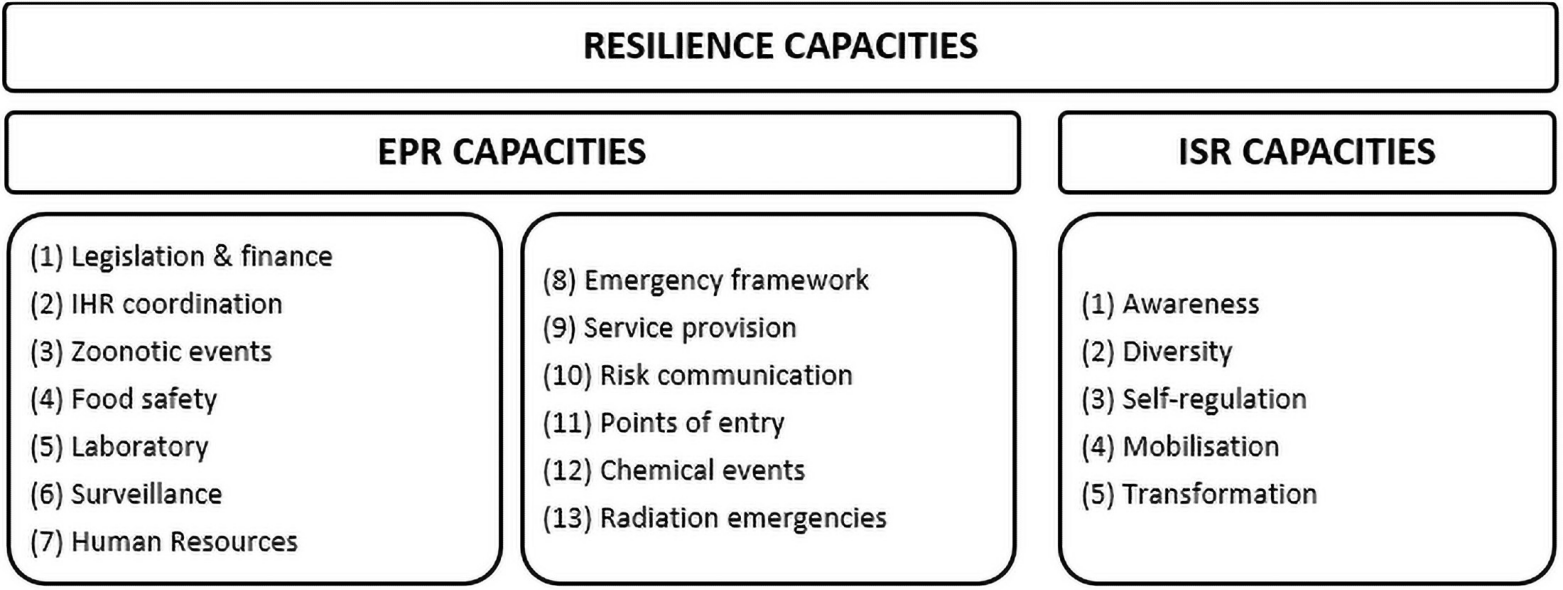
Capacities of health system resilience.

Based on the description of these capacities for both EPR and ISR, we extracted attributes for each capacity—see supplementary Appendix 2 (S2) in S1 File. A total of 18 components are identified: the five for ISR plus the thirteen for EPR (Fig 2).

The capacities of system resilience are mutually reinforcing, and ultimately work together to improve performance of health systems [47]. In the rest of this paper, we explore these capacities and their implications, applying this through an analysis of resilience across the countries of the WHO African Region.

## Methodology

To operationalize the different levels of resilience and its constituent capacities of EPR and ISR explored, we opt to develop an index for each. Indices minimize the focus on specific indicators, which may only provide partial perspectives of both capacities, and may become targets in of themselves [49, 50]. This methodological exploration seeks to generate empirical measures for resilience, aimed at supporting decision makers as they plan and monitor the levels of resilience they need in order to mitigate against the effects of shock events.

### Analytical approach

For EPR, the IHR SPAR scores for the thirteen capacities were used. The methodology for deriving this is standard and publicly available, with specific attributes defined for each capacity. Every country is scored against each attribute on a Likert scale ranging from 1–5 and a consolidated score generated as the Country IHR capacity index. This is reflected as ranging from 0–100 for the country.

For ISR, we replicated the above methodology to ensure comparability and minimize overlap. For each of the five inherent system resilience capacities, we derived attributes based on the descriptions from literature and expert consultations. These expert consultations were within a two-year (2016–18) period involving the 47 countries of the Region [46, 51]. Each attribute was then based on a Likert scale that scored from 1 to 5 with 1: nascent; 2: limited; 3: moderate; 4: developed; and 5: sustainable capacity. See supplementary Appendix 2 (S2) in S1 File for the full set of attributes.

### Data sources

For the ISR capacities, we opted for a cross-sectional facility-based data collection process, as the identified inherent system resilience attributes are actions most applicable at the unit of service provision. This is in line with the recognition that health facilities remain a weak link in the response to shocks, with an urgent need to understand core health service capabilities as a pre-requisite for system resilience [45]. A questionnaire was developed to score each of the attributes of the defined capacities for inherent system resilience. An attribute based questionnaire was opted for, in alignment with the IHR SPAR process, where the level of performance against each indicator is assessed using an attribute based scale [43, 52]. The questionnaire was incorporated into the routine surveillance system of the 47 countries of the region and aggregated using the regional Geographic Information Systems (GIS) Centre. The surveillance system is composed of field based technical experts who routinely visit health facilities as part of their work to conduct disease surveillance. They use standard questionnaires configured in open data kits (ODKs) and the information is captured using 2G mobile phones directly into the GIS system. The inherent system resilience tool was included amongst these tools to be compiled during their routine surveillance activities. The field team visits a facility, and together with the facility management team, review the constituent attributes. Together, they agree on a score against the Likert scale for the facility against the attribute.

The collective approach, and routine nature of the process is practical, as facilities are immediately aware of where their strengths and gaps are and can commence corrective action, without waiting for study results. Leveraging the routine facility surveillance process ensures that this is not a standalone, one-off exercise that provides no long-term benefit to the health facility. For the purposes of this assessment, we captured the information collected during the period– 1 December 2019 to 31 March 2020.

### Data consolidation for ISR

Each attribute was scored from 1 to 5 at the facility level. To derive the inherent system resilience capacity scores, the mean of responses was calculated as:

$$\mu_{x}=\sum_{i=1}^{n}p_{i}X_{i}=Y_{1}\times 1+Y_{2}\times 2+Y_{3}\times 3+Y_{4}\times 4+Y_{5}\times 5$$

where ***p***_***i***_ is the probability (relative frequency) of outcome ***X***_***i***_ (which ranges from 1 to 5), and ***Y***_***i***_ represents the frequencies of the specified outcomes.

A total of 5,294 facilities collected their information during this period out of the estimated 99,000 facilities in the region [53]. We applied a basic number of facilities that needed to have data in each country for it to have its average score calculated based on a cut off of at least fifteen primary care facilities, and/or 10% of primary care facilities in a country had reported—either option from at least half the sub national units in a country. As such, facilities were purposively included, but with a base criterion to ensure minimal bias from a single facility type, number or geographical location. Out of the forty-seven countries of the region, thirty-two had facilities reporting within the selected period and twenty-four met the inclusion criterion, as shown in Table 2.

**Table 2:** Number of health facilities assessing their inherent system resilience by country, December 2019–March 2020.

Country Name	Number of facilities reporting	Country Name	Number of facilities reporting	Country Name	Number of facilities reporting
Algeria	N.A	Eswatini	11	Namibia	32
Angola	60	Ethiopia	317	Niger	7
Benin	17	Gabon	12	Nigeria	241
Botswana	15	Gambia, The	N.A.	Rwanda	8
Burkina Faso	2	Ghana	717	Sao Tome and Principe	N.A.
Burundi	N.A.	Guinea	1	Senegal	N.A.
Cabo Verde	N.A.	Guinea-Bissau	10	Seychelles	N.A.
Cameroon	1040	Kenya	360	Sierra Leone	18
Central African Republic	51	Lesotho	N.A.	South Africa	19
Chad	229	Liberia	96	South Sudan	860
Comoros	N.A.	Madagascar	106	Togo	1
Congo, Dem. Rep.	489	Malawi	58	Uganda	92
Congo, Rep.	25	Mali	N.A.	United Republic of Tanzania	38
Cote d’Ivoire	63	Mauritania	N.A.	Zambia	253
Equatorial Guinea	N.A.	Mauritius	N.A.	Zimbabwe	48
Eritrea	N.A.	Mozambique	N.A.	**REGIONAL**	**5294**

*N. A: Countries where no facility data was collected.

For countries that did not meet the inclusion criteria, the inherent system resilience score estimate was imputed using a multivariate imputation via chained equations (MICE) methodology. This analysis was carried out using R software package. We specified an stochastic implicit imputation model per variable, to account for variation in unique observations, where predictions on country-specific values inherent system resilience were based on the other variables of health system functionality—the premise being the relationship between resilience and the other health system functionality variables is predictable [47]. See supplementary Appendix 3 (S3) in S1 File.

### Data consolidation and generation of indices

To achieve comparability with scores derived for ISR and EPR, country values across the various attributes were normalized and rescaled to a unitless range of 0–100 to establish comparability in terms of scale. The normalized values were determined using the formula:

$${(X}^{\prime }=\frac{\left(x_{i}-x_{Minimum}\right)}{\left(x_{Maximum}-x_{Minimum}\right)})\times 100$$

where x _minimum_ and x _maximum_ are the lowest and highest reported values. The arithmetic mean of the normalized values of each capacity was calculated. Thus, a score was generated for each capacity and an overall score computed, as the arithmetic mean of the scores of the sub-capacities.

The overall resilience index was then determined as the arithmetic mean of the EPR and ISR scores for each country.


$$Resilience\;Index=\frac{EPR\;Score+ISR\;Score}{2}$$

We opted not to equally weight each of the capacities of EPR and ISR as it would skew the results towards EPR, which is comprised of 13 capacities, against ISR, which is comprised of 5 capacities. We thus opted to compute the average at the level of the two operational functions of resilience, given their distinctness and complementarity for achieving overall resilience. Efforts in each country will thus be determined by their specific performance against these two core functions—the lower the score, the stronger the emphasis needed.

Finally, we computed bias-corrected non-parametric confidence intervals of the EPR and ISR sub-indices, as well as the overall resilience index, using 1000 bootstrap replicates, for a 95% confidence interval.

### Validating the index

The optimal metric for ascertaining validity of the computed index would be to assess its correlation with temporal variability in health service provision and coverage levels in reference to different shock events. This was however not feasible due to absence of data specifically on shock events severity and impact—the closest information being limited to numbers of reported disease events only [54]. We therefore explored alternative processes for ascertaining face validity of the computed index.

The assumptions that were validated included: (1) whether the EPR and ISR are actually measuring different aspects of resilience, and (2) whether resilience levels are associated in any way with health outcomes—as we postulate a resilient system should facilitate better health outcomes, keeping other contributors constant. Correlations were interpreted in a standard manner, as defined in literature: <0.35 low, 0.36–0.67 moderate, 0.68 to 1.0 as high [55].

Looking at whether there was any correlation between EPR and ISR, we computed a Pearsons correlation coefficient between the emerging scores of both across the forty-seven countries. A high, or even moderate correlation would suggest some overlap amongst the capacities being assessed while a low correlation would suggest low potential overlap. Similarly, we computed a correlation coefficient between the service provision capacity of the IHR (C.9), and the ISR, to explore potential overlap between this IHR core capacity, which explores certain aspects of preparedness of health systems to shocks [52].

Next, the association between the emerging resilience index and health outcomes across the forty-seven countries assessed was explored. Within the regional framework of actions for health systems development, resilience is conceptualized as an output level measure, that interacts together with access to essential services, quality of care and demand for services to produce the observed level of utilization of essential services [46, 51]. One would therefore expect a correlation between the emerging resilience index and universal health coverage (UHC) service outcome component. We explored this correlation comparing the values of the spearman’s rank coefficient for correlation between the calculated resilience index and the UHC service coverage components [56]. While we anticipate a correlation, we do not expect this to be high given the effects of other health system functionality components on UHC—such as access to and quality of care and demand for services [47, 48]. We further explored this relationship between UHC service coverage and the emerging resilience index by examining the changes in correlation of UHC with health system functionality with versus without the resilience index included [47]. The inclusion of the resilience index should increase the correlation between the UHC service coverage outcomes and the functionality index.

### Internal consistency and scale item reliability

Finally, we explored the internal consistency of the scale items that constitute each attribute of the inherent system resilience sub-index, by computing the Cronbach co-efficient alpha (c-alpha) of the twenty-nine (29) constituent attributes of the ISR sub-index. Further, we also computed c-alpha scores for each of the five components of ISR. Based on the c-alpha results, we determined the internal consistency and parsimony of the set of attributes used in the assessment of ISR. This is important as these attributes were extracted from literature and have not been tested before (see supplementary Appendix 3 (S3) in S1 File).

### Presenting the resilience index

We examined the resilience index and its constituent ISR and EPR components. We examined the overall resilience index by country, and the contribution of the two component sub-indices. This revealed, in each country, the drivers and magnitude of overall resilience.

### Patient and public involvement statement

Neither patients nor the public were directly involved in the design of the study as it was primarily analytical. The research question and measures were not informed by patients’ or public experiences, as this study was largely analytical and based on publicly available data.

## Results

### Construct validity

First, the calculated Pearson correlation coefficient between the ISR and EPR was r = 0.189. This is a low correlation, confirming discriminant validity and the assumption that EPR and ISR are different aspects of resilience. Second, the correlation between the IHR capacity C.9 (Health Service Provision), and the ISR was r = 0.149, suggesting that the EPR and the ISR monitor different aspects of overall health system resilience.

Third, the calculated spearman’s rho between the resilience index and the three UHC service coverage outcome components was r = 0.466, a moderate correlation. This suggests a relationship between the calculated resilience index and UHC service coverage, showing its value as an important contributor to UHC attainment. Similarly, the correlation between the UHC service coverage outcomes and the system functionality was strong when it included the computed resilience index (r = 0.7844, p<0.001), but reduced when the resilience was removed (r = 0.7056). The computed resilience index is thus contributing to system functionality that is needed for attaining UHC service outcomes. See supplementary Appendix 6 (S6) in S1 File.

### Internal consistency and scale item reliability

The constituent attributes of the ISR yielded a c-alpha coefficient of 0.991 which points to strong internal consistency of its attributes. Furthermore, c-alpha scores for the awareness, diversity, mobilization, self-regulation and transformation components of ISR were 0.899, 0.978, 0.857, 0.984 respectively. This validates the choice of scale items, and points to their relevance in assessing the overall construct. The strong alpha co-efficients for each of the components also validates the internal consistency of the specific scale items that make up the respective constructs. Their individual strengths are a good pointer of the internal consistency of the computed ISR sub-index. See supplementary Appendix 7 (S7 Table) in S1 File.

### Status of resilience in the WHO African Region

**Fig 3:**
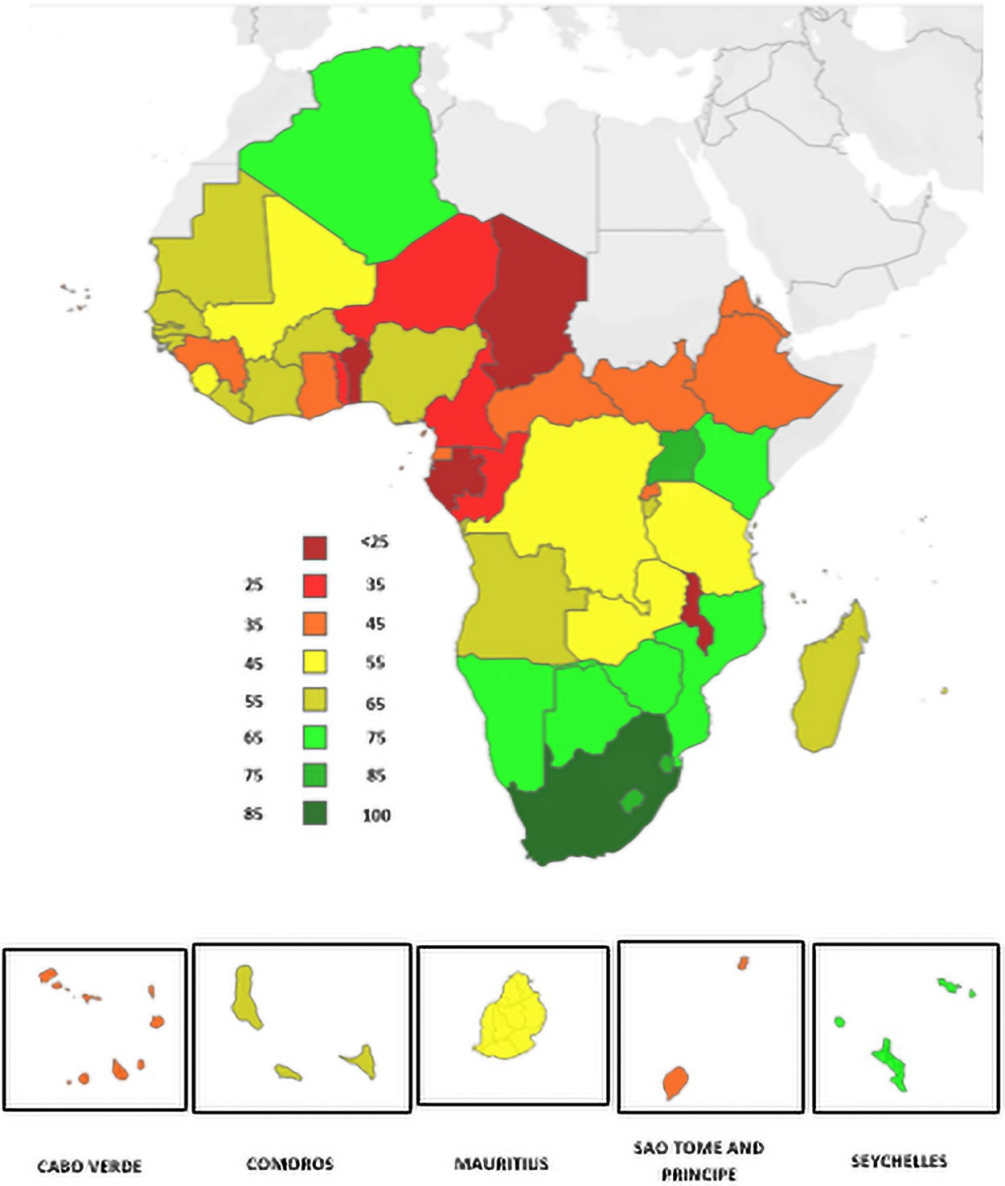
Relative resilience by country of the WHO African Region.

On confirming the face validity of the index, we now present the relative state of resilience in the region in Fig 3 below.

**Fig 4:**
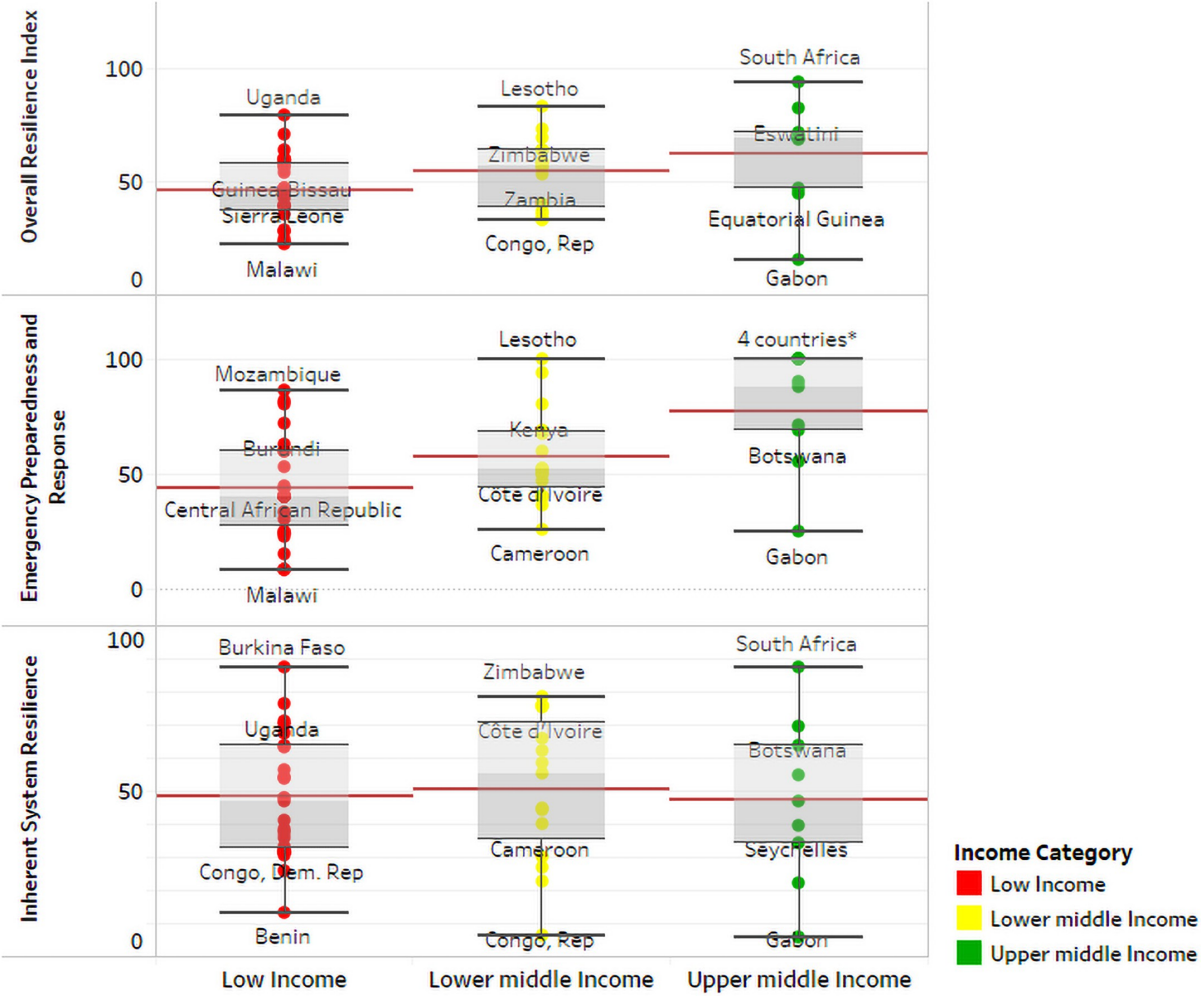
Distribution of inherent system resilience, emergency preparedness and response and overall resilience, by income group in the WHO African Region. *—Four countries with maximum EPR scores are Eswatini, Lesotho, Seychelles and South Africa. Countries displayed on figure represent maximum, 75^th^ percentile, 25^th^ percentile and minimum.

The findings show a variety of levels of resilience. The countries in the central belt of the African region appear to generally have lower levels of resilience. Comparing the relative resilience average for countries by income status, we see in Fig 4 (Fig 4) below that overall resilience is driven by income level—with this improving the higher the income group. However, this pattern is only seen with EPR and not with ISR.

Table 3 provides a country-specific overview of overall resilience and the ISR and EPR indices.

**Table 3:** Country specific values of overall resilience index and contribution of inherent system resilience and emergency preparedness and response scores.

Country	ISR score	EPR score (IHR SPAR 2018)	Overall Resilience Index
**Algeria**	54.7	88.0	71.4
**Angola**	22.6	94.0	58.3
**Benin**	13.4	33.0	23.2
**Botswana**	69.7	69.0	69.3
**Burkina Faso**	87.6	40.0	63.8
**Burundi**	47.7	72.0	59.9
**Cabo Verde**	26.9	47.0	37.0
**Cameroon**	40.0	26.0	33.0
**Central African Republic**	38.6	45.0	41.8
**Chad**	25.7	23.0	24.3
**Comoros**	44.5	69.0	56.8
**Congo, Dem. Rep**.	33.2	60.0	46.6
**Congo, Rep**.	6.3	60.0	33.2
**Cote d’Ivoire**	75.9	52.0	63.9
**Equatorial Guinea**	34.3	55.0	44.6
**Eritrea**	30.3	40.0	35.2
**Eswatini**	63.5	100.0	81.7
**Ethiopia**	46.8	40.0	43.4
**Gabon**	6.3	25.0	15.7
**Gambia, The**	56.3	63.0	59.7
**Ghana**	44.4	36.0	40.2
**Guinea**	70.2	8.0	39.1
**Guinea-Bissau**	71.2	43.0	57.1
**Kenya**	58.2	80.0	69.1
**Lesotho**	65.6	100.0	82.8
**Liberia**	31.6	80.0	55.8
**Madagascar**	62.9	53.0	58.0
**Malawi**	36.0	8.0	22.0
**Mali**	67.4	40.0	53.7
**Mauritania**	76.0	40.0	58.0
**Mauritius**	22.1	71.0	46.6
**Mozambique**	54.4	86.0	70.2
**Namibia**	46.9	90.0	68.5
**Niger**	41.1	15.0	28.0
**Nigeria**	62.1	50.0	56.0
**Rwanda**	53.9	25.0	39.4
**Sao Tome and Principe**	30.1	40.0	35.0
**Senegal**	75.1	52.0	63.5
**Seychelles**	39.6	100.0	69.8
**Sierra Leone**	31.7	60.0	45.8
**South Africa**	87.6	100.0	93.8
**South Sudan**	37.6	40.0	38.8
**Tanzania**	31.6	24.0	27.8
**Togo**	76.3	82.0	79.2
**Uganda**	63.9	30.0	47.0
**Zambia**	55.2	50.0	52.6
**Zimbabwe**	78.3	67.0	72.6
***Regional Average***	**42.9**	**47.6**	**48.4**
***Minimum value***	**5.8**	**8.0**	**15.4**
***25th percentile***	**32.4**	**40.0**	**39.0**
***75th percentile***	**64.8**	**71.5**	**63.9**
***Maximum value***	**87.6**	**100.0**	**93.8**
***Standard deviation***	**20.8**	**25.6**	**18.0**
***Standard Error***	**3.03**	**3.73**	**2.62**

The overall resilience index of 48.4 is rather low. This value varies significantly in the Region, with a wide range observed across countries. The minimum and maximum value are 15.4 and 93.8, with the difference between the 25^th^ and 75^th^ percentiles being 24.9 with a standard deviation of 18.0. ISR contributes less to overall resilience as compared to EPR– 42.9 versus 47.6, respectively, with the number of countries with lower ISR than the EPR, being 26 out of the 47. Overall resilience is highest in South Africa, Lesotho, Eswatini, Uganda and Zimbabwe. South Africa is the only country in the top 5 for both EPR and ISR. The high EPR scores in Eswatini and Lesotho drive their high overall score. Together with South Africa, the other countries with high ISR are Burkina Faso, Zimbabwe, Uganda and Mauritania. Some outlier countries include Rwanda and Ghana appear to have lower resilience than would be perceived—driven by their low EPR scores.

The overall resilience is lowest in Togo, Chad, Benin, Malawi and Gabon, with the low EPR driving this for Togo, Chad and Malawi. The countries with low ISR include Angola, Mauritius, Benin, Congo and Gabon. Table 4 provides a regional summary of ISR and its different constituent components.

**Table 4:** Regional summary of inherent system resilience capacities.

Parameter	*Awareness*	*Diversity*	*Self-regulation*	*Mobilization*	*Transformation*
**Regional Average**	**33.34**	**51.87**	**38.61**	**29.73**	**25.58**
**Minimum value**	**4.61**	**18.85**	**8.03**	**1.78**	**3.25**
**25th percentile**	**16.93**	**36.95**	**26.93**	**21.77**	**13.94**
**75th percentile**	**73.93**	**72.04**	**76.05**	**72.69**	**58.85**
**Standard deviation**	**29.92**	**23.85**	**30.37**	**31.07**	**28.44**

The capacity with the highest value is service diversity (51.1, standard deviation of 22.2), followed by self-regulation capacity (38.6 standard deviation of 29.6), awareness (33.3, standard deviation of 29.3), mobilisation (29.7, standard deviation of 31.0) and transformation (25.6, standard deviation of 28.1). The diversity component scores highest, while the transformation of systems scores lowest. We note that there is a wide variation in values for each of these components across all the countries in the region.

## Discussion

We have explored a methodological approach to making operational health system resilience, proposing the generation of an index for common measurement, planning and monitoring. The core output of this paper is the methodological exploration, which forms the basis around which countries can begin to strengthen data collection on elements of health system resilience. The index generated is an application of the proposed methodology, and presents the potential for decision makers to identify core areas for action at country level, towards building system resilience. Overall resilience is low, both when we look at capacities targeting specific shocks, and non-specific shocks. Understanding the effect of a shock on health services is incomplete if we only look at EPR capacities, as systems are constantly facing known and unknown shocks—not only disease and environmental, but also political and economic shocks.

This analysis shows the importance of two perspectives of resilience, which are equally relevant, given their differential focus and effects. As evidenced by the COVID-19 pandemic, severity of a shock in a country bears no singular correlation with reported IHR core capacities, as it was an unpredictable shock for which countries could not have built EPR capacities to respond to [57–59].

The finding that ISR is lower than EPR is reflected in reality, since a lot more emphasis has been placed on improving the latter. Countries that have experienced public health emergencies of epidemic threat such as Democratic Republic of Congo, Liberia and Sierra Leone have focused on strengthening their EPR actions, but have continued to have their systems overwhelmed when unpredictable shocks like COVID-19 arise. Investments in one dimension of resilience limits the ability to attain the twin goals of improved emergency preparedness and response (health security) and universal health coverage, a dichotomy which has been documented as being in ‘tension’ [60].

Moving forward, the COVID-19 pandemic has demonstrated that countries will increasingly face harder to predict shocks, increasing the need to invest in ISR [6]. As shown in this paper, this is not about embedding EPR actions into health system strengthening efforts, but rather building ISR as a core construct of a functioning system.

Actions to build ISR are taken from the community, to the national political level. Community resilience needs to be integrated together with the health system interventions plus political and social actions to establish the needed ISR in a system. The role of political commitment and strong sector governance in building resilience cannot be overlooked [61]. The call for strengthened multilateral political action to bring countries together for belter health security has been elaborated on numerous platforms, including though a global solidarity statement by Heads of States to strengthen national, regional and global capacities and resilience to future pandemics, in the context of COVID-19 [62].

From the literature, we note many of the actions that build inherent system resilience relate to ‘software’ in a system—the leadership styles, relationships amongst decision makers, and other elements difficult to define norms [9]. Given the focus on decision makers, it is important that the resilience assessment also provides some normative actions they can focus on. We propose some actions across different components of the health system that could improve ISR (see supplementary Appendix 8 in S1 File).

### Study limitations

We recognize there remain areas of further work to continue unpacking the concepts and application of resilience.

Firstly, we lack information on the actual impact of many shocks to fully package how to respond to them. We commonly focus on disease and environmental shocks effects on morbidity and mortality, but a more effective measure would focus on the way shocks influence the context, capacity and processes of the health system. The COVID-19 pandemic has highlighted this.

Second, resilience challenges are context-dependent, dynamic, and vary even within a country, or over time. The values we derive are limited in use to a national level decision maker and based on time-specific data. It would be worthwhile developing a dynamic index, which allows derivation of outputs over time and at different places. Inclusion of exogenous risk factors such as political fragility and other key influencers of health system resilience, will also strengthen the index.

Third, we recognize the potential challenges with using two data sets collected in different ways to generate the overall picture, including the variation in level of reporting. We made the effort to harmonize the methods as much as was possible and avoided overlaps in measurement. The data would benefit more from being collected at the same time and in the same way for all the attributes. We also note the limitation in using cross-sectional data in generation of aggregate estimates, as well as the weakness in their design for proving causality. However, in the absence of strong routine information systems across the region, this has formed the mainstay of generating estimates, coupled with modelled estimates, and some research has even corroborated findings from both data sources, noting their similarity in direction and magnitude. Estimates from cross-sectional population-based surveys are thus widely used in correlation analyses, and thus the authors’ decision to include this in the analysis, is aligned with available evidence.

Fourth, there remain widely documented limitations in the IHR core capacity scoring for countries, given that it is a self-reporting process that may avail itself to bias and reduced objectivity. These have been noted in the recently published World Health Assembly (WHA) 74 report on the review of the implementation of the International Health Regulations (2005). The report recommends a transition to a peer-reviewed system whereby countries review their capacities together with other countries, make the results public and, support one another, to provide a common accountability framework, supported by the WHO Secretariat. We propose improvements to the EPR index in this study once further recommendations on this methodology for IHR SPAR peer review become available from the WHO Secretariat.

Furthermore, to align with the methodology of the IHR, and recognizing the importance of assessing inherent system level resilience at the national level, that represents the level of system level governance in countries, is an area of improvement of this study. Currently, the proposed assessment of ISR is at facility level. In order to strengthen this process of monitoring health system resilience, there needs to be a complementary process at national level, incorporating information from key policy makers through expert Delphi-panels and key informant data collection processes. This national level process will strengthen the index, and supplement the data collection process at facility level.

Finally, we analyse and present information on resilience even when we argue it should be an integral construct of how a health system functions. By extracting and analysing it independently, we create a perception it can be targeted independently. Further research consolidating the resilience information into overall health system functionality is needed as a next step in advancing the evidence.

## Conclusion

We highlight in this paper a methodological exploration to build and monitor resilience against shock events, based on the existing conceptual understandings. We have explored the need to approach resilience not just as emergency preparedness and response activities, but also as a focus on inherent health system attributes. Both EPR and ISR capacities are important for resilience. We have also illustrated how these concepts can be measured and offered guidance on where each country in the WHO African Region needs to place emphasis to build its own resilience to shocks. The type and severity of shock events countries face are multiplying, making the need for a holistic approach to building resilience more urgent. Resilience cannot be perceived in a piecemeal fashion, as the COVID-19 pandemic has shown us, but rather needs to be built with a comprehensive focus on capacities that address both predictable, and unpredictable shock events.

## Supporting information

S1 File Overall publication supplementary appendix. (DOCX)
