# Supplementary material for: On the resilience of health systems: A methodological exploration across countries in the WHO African Region
Source: PLoS One. 2022 Feb 7;17(2):e0261904. doi: 10.1371/journal.pone.0261904 (PMC8820618; doi:10.1371/journal.pone.0261904)
Supplement: S1 File — (DOCX) [file pone.0261904.s001.docx]

**Supplementary Appendix**

**Background**

This supplement provides additional information to enable persons that would want to replicate the process leading to the results to do so. It highlights how the methods explained in the paper have been implemented using the data, eventually leading to the results. It follows a logical approach from the raw data to the eventual published results.

Data used for computing Emergency Preparedness and Response scores in each country is based on the 2018 IHR core capacities retrieved from the Electronic State Parties Self-Assessment Annual Reporting Tool and data on inherent system resilience is based on field responses from 5294 health facilities in the WHO Africa Region, captured through ODK based mobile technology, aggregated and transmitted through the WHO AFRICA Geographic Information Systems Centre.

**S1- List of countries in the WHO Africa Region by income classifications**

**S2- Capacities and their component attributes for Emergency Preparedness and Response (EPR) and Inherent System Resilience (ISR)**

**S3- Calculating Inherent Resilience Scores**

**S4-Calculating Overall Resilience Index**

**S5- Descriptive Statistics for EPR, ISR and Overall Resilience Index**

**S6- Index Validity**

**S7- Internal Consistency and Scale Reliability**

**S8- Suggested investments across the health system to contribute to resilience**

**S1- List of countries in the WHO Africa Region by income classifications**

**Table S1**

| **High / Upper Middle Income** | **Lower-middle Income** | **Low Income** |
| --- | --- | --- |
| Seychelles (High Income) | Angola | Benin |
| Algeria | Cameroon | Burkina Faso |
| Botswana | Cabo Verde | Burundi |
| Equatorial Guinea | Comoros | Central African Republic |
| Gabon | Congo, Rep. | Chad |
| Mauritius | Cote d'Ivoire | Congo, Dem. Rep. |
| Namibia | Eswatini | Eritrea |
| South Africa | Ghana | Ethiopia |
|  | Kenya | Gambia, The |
|  | Lesotho | Guinea |
|  | Mauritania | Guinea-Bissau |
|  | Nigeria | Liberia |
|  | Sao Tome and Principe | Madagascar |
|  | Senegal | Malawi |
|  | Zambia | Mali |
|  | Zimbabwe | Mozambique |
|  |  | Niger |
|  |  | Rwanda |
|  |  | Sierra Leone |
|  |  | South Sudan |
|  |  | Togo |
|  |  | Uganda |
|  |  | United Republic of Tanzania |

**S2- Capacities and their component attributes for Emergency Preparedness and Response, and Inherent System Resilience**

**ASSESSING ATTRIBUTES OF INHERENT SYSTEM RESILIENCE**

1. **Process of assessment**
   1. **Level of assessment**

Service provision units, which can be

- community units, assessing inherent system resilience in their specific community area
- Public, or non-public health facilities (all types) assessing the facility’s resilience
- Sub national management units like districts / county’s / states assessing their resilience
- National Ministries responsible for Health assessing overall resilience in the country
  1. **Process of assessment**

The team going to conduct the assessment, as part of routine surveillance processes, work together with facility management to deliberate on the score for the attribute

The scoring for each attribute ranges from 1 – 5.

**Nascent capacity:** None of the activities needed for the attribute are present

**Limited capacity:** Some activities needed for the attribute are present, but none are adequate

**Developing capacity:** Some activities needed for the attribute are present at adequate levels

**Developed capacity:** All activities needed for the attribute are present at adequate levels, but not domestically (government, local resources) financed

1. **Sustainable capacity:** All activities needed for the attribute are present at adequate levels, and are domestically (government, local resources) financed

If an attribute is not applicable at a given level of the system, then NA is captured. Do not leave any attribute unscored.

**Table S2. Inherent System Resilience capacities and their component attributes**

| **Capacity** | **Attributes** |
| --- | --- |
| Awareness | There is up to date (under 1 year old) data mapping the health system assets – HR, infrastructure, commodities – that can be mobilized in the event of a stress event |
|  | There is an up to date (under 1 year old) mapping of potential health risks at the lowest level of the health system – health center or community unit |
|  | There is a functional epidemiological surveillance network regularly (weekly) reporting on status of potential disease events |
|  | The health sector is conducting regular (at least annual) predictive modelling of major health risks facing different populations and sharing this information with concerned parties |
|  | The health sector is conducting simulation exercises (stress tests) against the 5 shock events of highest risk of occurrence |
| Diversity | Primary care facilities are providing at least 80% of the range of essential services they are expected to provide |
|  | Physical, financial and/or social barriers hindering access to available essential services are known and minimized |
|  | There is a clear strategy to scale up the provision of essential services currently not currently being provided |
|  | Health facilities have basic capacities needed for provision of a **broad range of essential services:**   - basic amenities: reliable power, water, sanitation, - basic equipment, - standard precautions for infection prevention, - diagnostic capacity, - essential medicines |
|  | Staff are appropriately skilled, and supervisory systems functional enough to identify rare / uncommon events when they occur |
| Self-Regulation | The primary care (front line) facilities have the needed technical capacity to identify and isolate a health threat |
|  | There are mechanisms at the management level supporting health facilities to target local resources to an identified health threat without need for bureaucratic authorizations |
|  | Health facilities are aware of, and able to put in place contingency mechanisms that allow continued essential service provision even when responding to a threat |
|  | Sources of additional capacities (HR) that may be needed to respond to the threat are identified and procedures to bring these on board are known & agreed |
| Mobilization | There are functional mechanisms for communication and engagement with non-public health partners working within the areas of responsibility of primary care facilities – such as private sector, NGOs, CSOs, and others |
|  | There are functional mechanisms for communication and engagement of primary care facilities with communities they are working within |
|  | There are functional mechanisms for communication and engagement with health related sectors working within the areas of responsibility of primary care facilities – such as agriculture, transport, education, and others |
|  | There are pre-agreed mechanisms for sharing of personnel, funds and capacities amongst stakeholders working within their areas of responsibility of primary care facilities |
| Transformation | There is regularly updated information on the state and performance of the health system |
|  | There are agreed protocols to guide absorption of resources and skills mobilized during a response to an event into the routine system |
|  | There are protocols to constantly monitor changing performance of the health system during a shock event |
|  | There is guidance on comprehensive recovery planning based on assessment, and investment across the health system |
|  | Process documentation and intelligence is planned and conducted during a shock event, to generate lessons |

**Table S3. Emergency Preparedness and Response-IHR core capacities and attributes**

| **Core Capacity** | **Attributes** |
| --- | --- |
| **National legislation, policy & financing** | Legislation, laws, regulations, administrative requirements, policies or other government instruments in place are sufficient for implementation of IHR. |
|  | Funding is available and accessible for implementing IHR NFP functions and IHR core capacity strengthening. |
| **Coordination and NFP communications** | A mechanism is established for the coordination of relevant sectors in the implementation of IHR. |
|  | IHR NFP functions and operations are in place as defined by the IHR (2005). |
| **Surveillance** | Indicator based, surveillance includes an early warning3 function for the early detection of a public health event. |
|  | Event based surveillance is established. |
| **Response** | Public health emergency response mechanisms are established. |
|  | Case management procedures are implemented for IHR relevant hazards. |
|  | Infection prevention and control (IPC) is established at national and hospital levels. |
|  | A programme for disinfection, decontamination and vector4 control is established |
| **Preparedness** | A Multi-hazard National Public Health Emergency Preparedness and Response Plan is developed. |
|  | Priority public health risks and resources are mapped |
| **Risk communication** | Mechanisms for effective risk communication during a public health emergency are established. |
| **Human resource capacity** | Human resources available to implement IHR core capacity requirements |
| **Laboratory** | Coordinating mechanism for laboratory services is established. |
|  | Laboratory services are available to test for priority health threats |
|  | Influenza surveillance is established. |
|  | System for collection, packaging and transport of clinical specimens is established |
|  | Laboratory biosafety and laboratory biosecurity (Biorisk management1 ) practices are in place. |
|  | Laboratory data management and reporting is established |
| **Points of Entry** | General obligations at PoE are fulfilled. |
|  | Coordination in the prevention, detection and response to public health emergencies at PoE is established. |
|  | Effective surveillance and other routine capacities is established2 |
|  | Effective response at PoE is established. |
| **IHR Potential hazard 1: zoonotic events** | Mechanisms for detecting and responding to zoonoses and potential zoonoses are established. |
| **IHR Potential hazard 2: food safety** | Mechanisms are established for detecting and responding to foodborne disease and food contamination. |
| **IHR Potential hazard 3: chemical events** | Mechanisms are established for the detection, alert and response to chemical emergencies. |
| **IHR Potential hazard 4: radiation emergencies** | Mechanisms are established for detecting and responding to radiological and nuclear emergencies. |

*A checklist, including the meta data which explains all the composite core capacities and their respective indicators is provided in the WHO Checklist and indicators for monitoring progress in the development of IHR core capacities in States Parties^1^.*

**S3- Calculating Inherent System Resilience Scores**

1. **Computing scores for Inherent System Resilience capacities**

To derive the scores for the inherent system resilience capacities, the mean of responses provided form facilities on the standard 5-category Likert-scale, was calculated as a measure of dispersion of the responses. The mean was calculated by:

$$\boldsymbol{\mu}_{\boldsymbol{x}}\boldsymbol{=}\sum_{\boldsymbol{i=1}}^{\boldsymbol{n}} \boldsymbol{p}_{\boldsymbol{i}}\boldsymbol{X}_{\boldsymbol{i}}\boldsymbol{=}\boldsymbol{Y}_{\boldsymbol{1}}\boldsymbol{\times1+}\boldsymbol{Y}_{\boldsymbol{2}}\boldsymbol{\times2+}\boldsymbol{Y}_{\boldsymbol{3}}\boldsymbol{\times3+}\boldsymbol{Y}_{\boldsymbol{4}}\boldsymbol{\times4+}\boldsymbol{Y}_{\boldsymbol{5}}\boldsymbol{\times5}$$

where $\boldsymbol{p}_{\boldsymbol{i}}$ is the probability (relative frequency) of outcome $\boldsymbol{X}_{\boldsymbol{i}}$ (which ranges from 1 to 5), and $\boldsymbol{Y}_{\boldsymbol{i}}$ represents the frequencies of the specified outcomes

1. **Data Imputation Methodology**

We implemented Multivariate Imputation by Chained Equations (MICE) using R software to impute for the missing data^2^, the methodology works under the assumption that given the variables we used in the imputation procedure (access, demand, quality and resilience), the missing data are missing at random (MAR). This implies that the probability that a value is missing depends only on observed values and not on unobserved values ^2–4^.

We used a regression equation with the relationship that overall performance is explained by the variables access, demand, quality and resilience. Based on this relationship, the missing variables were imputed using the predictive mean matching (PMM) a procedure implemented within the MICE package in R software. PMM produces imputed values that resemble the observed values better than methods based on the normal distribution^5^. This methodology ensures that if the original variable is right-skewed, PMM will then produce imputed values that follow the same distributional pattern.

With the imputation procedure we implemented we generated m=5,000 complete datasets from which we proceeded to estimate the regression model parameters on each of the m datasets and combine the estimates to one combined result which is the resulting final dataset which was utilized in the analysis.

1. **Deriving Overall Scores for Inherent System Resilience**

The inherent system resilience score per country was obtained by calculating the arithmetic mean of the values of its composite capacities. The mean was calculated by:

$$\boldsymbol{\mu}_{\boldsymbol{x}}\boldsymbol{=}\frac{\sum\boldsymbol{x}_{\boldsymbol{i}}}{\boldsymbol{n}}$$

1. **Normalizing inherent system resilience scores**

The two composite vital signs of the overall resilience index- EPR scores and ISR have different scales, thereby making it difficult to consolidate them. We therefore normalized all the ISR scores to a scale of 0 and 100, to make it comparable to the emergency preparedness and response scores. The normalized values were calculated using the formula:

$$\boldsymbol{(X}^{\boldsymbol{'}}\boldsymbol{=}\frac{\left( \boldsymbol{x}_{\boldsymbol{i}}\boldsymbol{-}\boldsymbol{x}_{\boldsymbol{Minimum}} \right)}{\boldsymbol{(}\boldsymbol{x}_{\boldsymbol{Maximum}}\boldsymbol{-}\boldsymbol{x}_{\boldsymbol{Minimum}}\boldsymbol{)}}\boldsymbol{)\times100}$$

**Table S4.** Country values for inherent system resilience capacities

| **Country** | **Awareness** | **Diversity** | **Self-Regulation** | **Mobilization** | **Transformation** | **ISR Score** |
| --- | --- | --- | --- | --- | --- | --- |
| Algeria | 9.90 | 40.48 | 73.11 | 73.62 | 58.85 | 54.73 |
| Angola | 23.95 | 35.74 | 25.38 | 18.15 | 10.23 | 22.62 |
| Benin | 9.90 | 40.48 | 13.40 | 1.78 | 3.25 | 13.40 |
| Botswana | 59.41 | 93.00 | 57.40 | 83.11 | 41.91 | 69.68 |
| Burkina Faso | 76.10 | 96.87 | 100.00 | 100.00 | 100.00 | 87.60 |
| Burundi | 23.95 | 18.85 | 73.11 | 73.62 | 58.85 | 47.72 |
| Cabo Verde | 58.37 | 74.21 | 8.03 | 2.15 | 3.25 | 26.95 |
| Cameroon | 37.68 | 49.68 | 34.16 | 40.52 | 32.10 | 39.96 |
| Central African Republic | 45.13 | 36.31 | 31.98 | 38.44 | 37.86 | 38.64 |
| Chad | 32.84 | 32.09 | 20.99 | 25.39 | 17.65 | 25.66 |
| Comoros | 87.03 | 38.11 | 28.49 | 25.39 | 45.67 | 44.55 |
| Congo, Dem. Rep | 39.35 | 36.95 | 28.49 | 35.26 | 24.72 | 33.22 |
| Congo, Rep | 9.90 | 18.85 | 8.03 | 1.78 | 4.69 | 6.34 |
| Côte d'Ivoire | 85.95 | 74.21 | 73.11 | 72.69 | 62.18 | 75.88 |
| Equatorial Guinea | 38.88 | 74.21 | 38.55 | 18.15 | 3.25 | 34.27 |
| Eritrea | 76.10 | 49.56 | 20.99 | 2.15 | 10.23 | 30.32 |
| Eswatini | 4.61 | 66.30 | 78.99 | 72.69 | 69.54 | 63.47 |
| Ethiopia | 55.21 | 51.70 | 41.17 | 43.04 | 38.05 | 46.81 |
| Gabon | 4.61 | 18.85 | 8.03 | 2.15 | 3.25 | 5.80 |
| Gambia | 9.90 | 31.06 | 100.00 | 72.69 | 58.85 | 56.33 |
| Ghana | 46.07 | 49.56 | 41.04 | 46.96 | 33.09 | 44.35 |
| Guinea | 9.90 | 74.21 | 87.18 | 92.75 | 62.18 | 70.24 |
| Guinea-Bissau | 4.61 | 93.00 | 78.99 | 92.75 | 58.85 | 71.17 |
| Kenya | 58.37 | 62.43 | 54.84 | 60.95 | 45.67 | 58.24 |
| Lesotho | 4.61 | 32.09 | 100.00 | 100.00 | 69.54 | 65.60 |
| Liberia | 38.88 | 31.06 | 28.99 | 35.12 | 23.78 | 31.62 |
| Madagascar | 60.84 | 66.30 | 61.82 | 58.48 | 55.63 | 62.94 |
| Malawi | 33.12 | 38.11 | 30.80 | 57.22 | 18.91 | 36.04 |
| Mali | 60.84 | 69.86 | 63.11 | 58.48 | 69.54 | 67.44 |
| Mauritania | 9.90 | 67.88 | 87.18 | 83.11 | 100.00 | 76.05 |
| Mauritius | 76.10 | 36.95 | 8.03 | 1.78 | 3.25 | 22.11 |
| Mozambique | 38.88 | 69.86 | 78.99 | 58.48 | 17.65 | 54.39 |
| Namibia | 36.58 | 69.22 | 38.55 | 57.24 | 25.00 | 46.93 |
| Niger | 100.00 | 96.87 | 13.40 | 1.78 | 3.25 | 41.08 |
| Nigeria | 76.37 | 57.06 | 49.33 | 70.41 | 50.79 | 62.07 |
| Rwanda | 9.90 | 35.74 | 100.00 | 38.54 | 66.42 | 53.87 |
| Sao Tome and Principe | 87.03 | 40.48 | 13.40 | 18.15 | 3.25 | 30.06 |
| Senegal | 9.90 | 62.43 | 78.99 | 92.75 | 100.00 | 75.07 |
| Seychelles | 85.95 | 74.21 | 20.99 | 18.15 | 4.69 | 39.62 |
| Sierra Leone | 41.90 | 27.56 | 29.41 | 33.63 | 26.05 | 31.65 |
| South Africa | 71.76 | 100.00 | 87.18 | 92.75 | 66.42 | 87.60 |
| South Sudan | 41.80 | 40.61 | 32.43 | 38.54 | 31.40 | 37.61 |
| Togo | 76.10 | 74.21 | 8.03 | 2.15 | 10.23 | 31.62 |
| Uganda | 100.00 | 74.24 | 68.43 | 72.14 | 58.85 | 76.33 |
| United Republic of Tanzania | 57.51 | 67.88 | 63.11 | 64.53 | 54.26 | 63.94 |
| Zambia | 58.66 | 60.92 | 50.17 | 58.45 | 40.37 | 55.15 |
| Zimbabwe | 87.03 | 69.86 | 78.99 | 73.62 | 69.54 | 78.28 |

**S4-Calculating Overall Resilience Index**

To derive the country-specific overall resilience index scores, an arithmetic mean, of country inherent system resilience scores and emergency preparedness and response score (IHR core capacity scores), was calculated, using the formula:

$$\boldsymbol{\mu}_{\boldsymbol{x}}\boldsymbol{=}\frac{\sum\boldsymbol{x}_{\boldsymbol{i}}}{\boldsymbol{n}}$$

**Table S5.** Country values for Emergency Preparedness and Response, Inherent System Resilience and Overall Resilience Index

| **Country** | **Inherent System Resilience Score** | **Emergency Preparedness and Response (IHR Core Capacity )** | **Overall Resilience Index** |
| --- | --- | --- | --- |
| Algeria | 54.73 | 88.00 | 71.37 |
| Angola | 22.62 | 94.00 | 58.31 |
| Benin | 13.40 | 33.00 | 23.20 |
| Botswana | 69.68 | 69.00 | 69.34 |
| Burkina Faso | 87.60 | 40.00 | 63.80 |
| Burundi | 47.72 | 72.00 | 59.86 |
| Cabo Verde | 26.95 | 47.00 | 36.97 |
| Cameroon | 39.96 | 26.00 | 32.98 |
| Central African Republic | 38.64 | 45.00 | 41.82 |
| Chad | 25.66 | 23.00 | 24.33 |
| Comoros | 44.55 | 69.00 | 56.77 |
| Congo, Dem. Rep | 33.22 | 60.00 | 46.61 |
| Congo, Rep | 6.34 | 60.00 | 33.17 |
| Côte d'Ivoire | 75.88 | 52.00 | 63.94 |
| Equatorial Guinea | 34.27 | 55.00 | 44.63 |
| Eritrea | 30.32 | 40.00 | 35.16 |
| Eswatini | 63.47 | 100.00 | 81.74 |
| Ethiopia | 46.81 | 40.00 | 43.40 |
| Gabon | 5.80 | 25.00 | 15.40 |
| Gambia | 56.33 | 63.00 | 59.67 |
| Ghana | 44.35 | 36.00 | 40.18 |
| Guinea | 70.24 | 8.00 | 39.12 |
| Guinea-Bissau | 71.17 | 43.00 | 57.09 |
| Kenya | 58.24 | 80.00 | 69.12 |
| Lesotho | 65.60 | 100.00 | 82.80 |
| Liberia | 31.62 | 80.00 | 55.81 |
| Madagascar | 62.94 | 53.00 | 57.97 |
| Malawi | 36.04 | 8.00 | 22.02 |
| Mali | 67.44 | 40.00 | 53.72 |
| Mauritania | 76.05 | 40.00 | 58.02 |
| Mauritius | 22.11 | 71.00 | 46.55 |
| Mozambique | 54.39 | 86.00 | 70.19 |
| Namibia | 46.93 | 90.00 | 68.47 |
| Niger | 41.08 | 15.00 | 28.04 |
| Nigeria | 62.07 | 50.00 | 56.03 |
| Rwanda | 53.87 | 25.00 | 39.43 |
| Sao Tome and Principe | 30.06 | 40.00 | 35.03 |
| Senegal | 75.07 | 52.00 | 63.53 |
| Seychelles | 39.62 | 100.00 | 69.81 |
| Sierra Leone | 31.65 | 60.00 | 45.83 |
| South Africa | 87.60 | 100.00 | 93.80 |
| South Sudan | 37.61 | 40.00 | 38.80 |
| Togo | 31.62 | 24.00 | 27.81 |
| Uganda | 76.33 | 82.00 | 79.16 |
| United Republic of Tanzania | 63.94 | 30.00 | 46.97 |
| Zambia | 55.15 | 50.00 | 52.58 |
| Zimbabwe | 78.28 | 67.00 | 72.64 |

**S5- Descriptive Statistics for EPR, ISR and Overall Resilience Index**

**Table S6**. Means, Variance and Standard Deviation Scores

| *Descriptive statistic* | Inherent System Resilience data value | EPR data value | Resilience index data value |
| --- | --- | --- | --- |
| *Count* | 47 | 47 | 47 |
| *Mean* | 48.84167 | 54.70213 | 51.7719 |
| *Mean LCL* | 42.73659 | 47.18403 | 46.49867 |
| *Mean UCL* | 54.94674 | 62.22022 | 57.04512 |
| *Variance* | 432.35171 | 655.64847 | 322.55826 |
| *Standard Deviation* | 20.79307 | 25.60563 | 17.95991 |
| *Mean Standard Error* | 3.03298 | 3.73497 | 2.61972 |
| *Coefficient of Variation* | 0.42572 | 0.46809 | 0.3469 |
|  |  |  |  |
| *Minimum* | 6.33865 | 8 | 15.67 |
| *Maximum* | 87.6 | 100 | 93.7977 |
| *Range* | 81.26135 | 92 | 78.1277 |
|  |  |  |  |
| *Median* | 46.93252 | 52 | 53.72088 |
| *Median Error* | 0.55447 | 0.68281 | 0.47892 |
| *Percentile 25% (Q1)* | 32.43451 | 40 | 38.96339 |
| *Percentile 75% (Q3)* | 64.77085 | 71.5 | 63.86917 |
| *IQR* | 32.33635 | 31.5 | 24.90578 |
| *MAD (Median Absolute Deviation)* | 11.3116 | 28 | 15.40118 |
| *Coefficient of Dispersion (COD)* | 0.37127 | 0.40221 | 0.27682 |
|  |  |  |  |
| *Mean Deviation* | 17.51291 | 21.1598 | 14.94667 |
| *Second Moment* | 423.15274 | 641.69851 | 315.69532 |
| *Third Moment* | -523.23434 | 3,287.5868 | 482.89514 |
| *Fourth Moment* | 397,987.99398 | 889,870.64627 | 239,134.16807 |
|  |  |  |  |
| *Sum* | 2,295.55826 | 2571 | 2,433.27913 |
| *Sum Standard Error* | 142.5501 | 175.54338 | 123.12692 |
| *Total Sum Squares* | 132,007.06674 | 170799 | 140,813.15499 |
| *Adjusted Sum Squares* | 19,888.1788 | 30,159.82979 | 14,837.67991 |
|  |  |  |  |
| *Geometric Mean* | 43.02222 | 47.55283 | 48.37194 |
| *Harmonic Mean* | 34.25182 | 38.25347 | 44.56358 |
| *Mode* | #N/A | 40 | #N/A |
|  |  |  |  |
| *Skewness* | -0.06011 | 0.20225 | 0.08609 |
| *Skewness Standard Error* | 0.33912 | 0.33912 | 0.33912 |
| *Kurtosis* | 2.22267 | 2.16105 | 2.39941 |
| *Kurtosis Standard Error* | 0.63715 | 0.63715 | 0.63715 |
| *Fisher Skewness* | -0.06211 | 0.20898 | 0.08895 |
| *Fisher Kurtosis* | -0.72744 | -0.79616 | -0.53035 |

**S6- Index Validity**

Pearson correlation coefficients were calculated to assess the associations between the Resilience Index and key measures for service continuity (UHC Index) and pandemic preparedness (Global Health Security Index), where the correlation co-efficient r, is derived by:

$$r=\frac{n(\sum xy)-(\sum x)(\sum y)}{\sqrt{[n\sum x^{2}-(\sum{x)}^{2}][n\sum y^{2}-(\sum{y)}^{2}}}$$

| ***Variable vs. Variable*** | ***R*** | ***R-Standard Error*** | ***t*** | ***p-value (two-tailed)*** |
| --- | --- | --- | --- | --- |
| overall_resilience_index vs. ihr_capacity | 0.82279 | 0.00718 | 9.71159 | 1.29021E-12 |
| overall_resilience_index vs. inherent_system _resilience_score | 0.71426 | 0.01089 | 6.84601 | 1.7246E-8 |
| global_health_security vs. overall_resilience_index | 0.43502 | 0.01802 | 3.24093 | 0.00224 |
| uhc_service_coverage vs. overall_resilience_index | 0.43066 | 0.0181 | 3.20105 | 0.00251 |
| EPR (ihr_capacity) vs. inherent system resilience_score | 0.18992 | 0.02142 | 1.29763 | 0.20103 |

**S7- Internal Consistency and Scale Reliability**

To assess internal consistency of the scale items that constitute the construct of inherent system resilience, we calculated cronbach alpha coefficients for the inherent system resilience index, as well as for the 5 constituent capacities. Cronbach alphas are derived using the formula:

$\boldsymbol{\alpha=}\frac{\mathbf{k}}{\mathbf{k-1}}\mathbf{*(1-}\frac{\sum\boldsymbol{\sigma}_{\mathbf{i}}^{\mathbf{2}}}{\boldsymbol{\sigma}_{\mathbf{x}}^{\mathbf{2}}}$ **)**

Where k is the number of items on the measure, $\sigma_{i}^{2}$ is the variance of each individual, and $\sigma_{x}^{2}$ is the variance of the total of all the items on the index. The results were as follows

|  | k | sum of variances | variance of total score | alpha score |
| --- | --- | --- | --- | --- |
| **Inherent System Resilience Capacity** | **24** | **11.69309524** | **266.3095372** | **0.990238236** |
| Awareness | 5 | 1.817096956 | 6.470494324 | 0.898964811 |
| Diversity | 6 | 2.387494023 | 12.91113217 | 0.978099024 |
| Mobilization | 4 | 1.740281081 | 6.170336707 | 0.957280148 |
| Self-Regulation | 4 | 1.46509881 | 5.481052621 | 0.976930063 |
| Transformation | 5 | 2.267036949 | 10.65682601 | 0.984086286 |

**S8- Suggested investments across the health system to contribute to resilience**

| **System building block** | **Proposed actions across inherent system resilience components** | | | | |  |
| --- | --- | --- | --- | --- | --- | --- |
|  | *Awareness* | *Diversity* | *Self-regulation* | *Mobilization* | *Transformation* |  |
| **Health workforce** | Up to date (under 1 year old) mapping of staff and their skills | Regular updating of staff trainings on new therapeutics | Staff with epidemiology skills at health facilities  Capacity building for workforce leadership on need for EPR and ISR capacities | Plan for surge capacity for staff (who, where from, how to bring them) |  |  |
| **Health products** | Up to date (under 1 year old) mapping of common supportive drugs & supplies e.g. Oxygen | Supplies readiness with assured supplies for commonly used supportive drugs and supplies e.g. Oxygen, PPEs, analgesics | Demand driven procurement system | Plan for surge capacity for drugs and supplies (which, where from, how to bring them) |  |  |
| **Health infrastructure** | Up to date (under 1-year-old) mapping of supportive infrastructure & equipment  Develop and make widely available Standard Operating Procedures for facility maintenance and up-keep to ensure readiness | Infrastructure readiness for essential service provision with water, sanitation and power services | Devolved authority for infrastructure mobilization | Plan for surge capacity for infrastructure (what, where from, how to bring it) |  |  |
| **Health information** | Event based and system capacity surveillance network  Up to date (under 1 year old) mapping of potential disease, environmental, economic and security risks & mitigation measures | Real-time monitoring of essential service provision |  | Established repository of health assets with in the area of responsibility | Documentation of outbreak response actions  Regular assessment of system functionality |  |
| **Service delivery** | Regular stress tests against different shocks | Multiple service delivery approaches to expand essential services provided | Protocols to ensure continued essential services provision in place |  | Continuous re-engineering of service delivery approaches and resource deployment |  |
| **Governance and coordination** | Standard operating procedures for deployment of staff, infrastructure and supplies in a shock event | Micro planning to manage service provision for hard to reach populations | Devolved authority to allow facility level decision making in emergencies | Engagement fora with communities, private and other sectors | Continuous medical education involving learning from past events  Standard recovery planning approach |  |
| **Financial management** |  |  | Authority and protocols for emergency spending | Establish local emergency fund with local stakeholders |  |  |

**References**

1. Checklist and indicators for monitoring progress in the development of IHR core capacities in States Parties [Internet]. World Health Organization; 2011 Feb. Report No.: WHO/HSE/IHR/2010.1.Rev.1. Available from: https://www.who.int/ihr/IHR_Monitoring_Framework_Checklist_and_Indicators.pdf

2. van Buuren S, Groothuis-Oudshoorn K. mice: Multivariate Imputation by Chained Equations in R. J Stat Soft [Internet]. 2011 Dec;45(3). Available from: https://www.jstatsoft.org/article/view/v045i03

3. Raghunathan TE, Lepkowski JM, Hoewyk JV, Solenberger P. A Multivariate Technique for Multiply Imputing Missing Values Using a Sequence of Regression Models. Survey Methodology. 2001;(12):11.

4. Schafer J, Graham J. Missing Data: Our View of the State of the Art. Psychological Methods. 2002 Jun 1;7:147–77.

5. White IR, Royston P, Wood AM. Multiple imputation using chained equations: Issues and guidance for practice. Statistics in Medicine. 2011;30(4):377–99.

6. Electronic State Parties Self-Assessment Annual Reporting Tool (e-SPAR) [Internet]. WHO e-SPAR. Available from: https://extranet.who.int/e-spar

7. Cabore JW, Karamagi HC, Kipruto H, Asamani JA, Droti B, Seydi ABW, et al. The potential effects of widespread community transmission of SARS-CoV-2 infection in the World Health Organization African Region: a predictive model. BMJ Global Health. 2020 May 1;5(5):e002647.
